# The Immunoexpression of Glucocorticoid Receptors in Breast Carcinomas, Lactational Change, and Normal Breast Epithelium and Its Possible Role in Mammary Carcinogenesis

**DOI:** 10.1155/2017/1403054

**Published:** 2017-11-19

**Authors:** Raja Alyusuf, Javed Fayyaz Wazir, Urmil Prabha Brahmi, Abdul Rahman Fakhro, Moiz Bakhiet

**Affiliations:** ^1^Department of Pathology, Salmaniya Medical Complex and Royal College of Surgeons in Ireland, Manama, Bahrain; ^2^Department of Pathology, Southend University Hospital, Prittlewell Chase, Westcliff-on-Sea, Essex SS0 0RY, UK; ^3^Department of Pathology, Arabian Gulf University, Manama, Bahrain; ^4^Fakhro Medical City, Manama, Bahrain; ^5^Department of Molecular Medicine, Arabian Gulf University, Manama, Bahrain

## Abstract

The role of estrogen and progesterone receptors in breast cancer biology is well established. In contrast, other steroid hormones are less well studied. Glucocorticoids (GCs) are known to play a role in mammary development and differentiation; thus, it is of interest to attempt to delineate their immunoexpression across a spectrum of mammary epithelia.* Aim*. To delineate the distribution pattern of glucocorticoid receptors (GRs) in malignant versus nonmalignant epithelium with particular emphasis on lactational epithelium.* Materials and Methods*. Immunohistochemistry (IHC) for GRs was performed on archival formalin-fixed paraffin-embedded tissue blocks of 96 cases comprising 52 invasive carcinomas, 21 cases with lactational change, and 23 cases showing normal mammary tissue histology.* Results*. Results reveal an overexpression of GRs in mammary malignant epithelium as compared to both normal and lactational groups individually and combined. GR overexpression is significantly more pronounced in HER-2-negative cancers.* Discussion*. This is the first study to compare GR expression in human lactating epithelium versus malignant and normal epithelium. The article discusses the literature related to the pathobiology of GCs in the breast with special emphasis on breast cancer.* Conclusion*. The lactational epithelium did not show overexpression of GR, while GR was overexpressed in mammary NST (ductal) carcinoma, particularly HER-2-negative cancers.

## 1. Introduction

Glucocorticoid receptors (GRs) are expressed in about 50% of invasive breast cancers and many breast cancer cell lines [1, 2]. Glucocorticoids (GCs) are known to play a role in mammary development and differentiation as well as an essential role in embryonic development and tissue homeostasis. They possess important anti-inflammatory and immunosuppressive properties [1, 3, 4].

The endocrine system coordinates the development of the mammary gland with reproductive development and the demand of the offspring for milk. Reproductive hormones—estrogen and progesterone—act directly on the mammary gland to bring about developmental changes.

Massive tissue remodeling occurs within the mammary gland during pregnancy. This results in the formation of the secretory lobuloalveolar units in preparation for lactation. Prolactin and progesterone are implicated in the initial proliferative phase of alveolar morphogenesis. Other hormones such as growth hormone and placental lactogen can influence alveolar morphogenesis [5]. The secretory activation stage of mammary gland development occurs after parturition and converts inactive lobuloalveoli to active milk secretion. This process is triggered by progestin withdrawal and depends upon augmented prolactin signaling [6]. Prolactin induces mammary gland development and lactogenesis. Binding of prolactin to its receptor leads to the phosphorylation and activation of Signal Transducers and Activators of Transcription (STAT) proteins, which in turn promote the expression of specific genes and are essential for mammopoietic and lactogenic signaling [7].

Metabolic hormones such as GCs, whose main role is to regulate metabolic responses to nutrient intake or stress, often have direct effects on the mammary gland as well. An understanding of the mechanisms by which hormones such as GCs bring about secretory and lactational differentiation may offer clues to the prevention of breast cancer as studies have linked full-term pregnancies in early life with reduction of the likelihood of breast carcinogenesis [8].

Mammary epithelial cells do not attain full differentiation until the advent of pregnancy. With the establishment of lactation, mammary epithelial cells undergo further differentiation [9, 10]. Hence, a mammary cell differentiation spectrum includes lactational change epithelium representing the most differentiated cell on the one hand and malignant epithelium representing the least differentiated cell on the other hand, with normal “resting” epithelium in between.

It is therefore of interest to answer the following questions: Since GCs are one of the hormones involved in the process of lactogenesis resulting eventually in terminal differentiation of the cell (lactational change), is the opposite true? Do they protect the cell from moving towards the least differentiated end of the spectrum (malignant change)?

Our aim is to study the immunoexpression of GRs along the aforementioned differentiation spectrum which may shed some light on the influence of GCs on carcinogenesis, if any.

In addition, we aim to study the relationship of GR expression with grade, estrogen receptor (ER), progesteron receptor (PR), and human epidermal growth factor receptor-2 (HER-2) expression status and axillary lymph node (ALN) status within the malignant group.

## 2. Materials and Methods

A retrospective study was performed on archival formalin-fixed paraffin-embedded tissue blocks retrieved from files of the Histopathology Department, Salmaniya Medical Complex, Kingdom of Bahrain, between 2001 and 2007.

A total of 96 cases were included in this study: 52 in the malignant group (invasive no special type (NST) ductal carcinomas) and 44 in the nonmalignant group (21 cases with lactational change and 23 cases with normal mammary tissue histology). The lactational change cases were compiled over time in our department. The method of compilation was that a representative block was kept from cases that show lactational change during routine practice. These were kept aside for future studies including this study. No accompanying clinical information was available. The reason for this approach was that lactational change is not routinely coded by Systematized Nomenclature of Medicine (SNOMED) by practicing pathologists, and it would have been difficult to retrieve retrospectively from the archives of the department by SNOMED diagnosis code.

Both normal and lactational groups comprised the nonmalignant group (44 cases) and were all taken from excisions performed for reduction mammoplasties or benign pathology. The latter comprised fibrocystic changes of the nonproliferative type, fibroadenomata, sclerosing adenoses, and inflammatory conditions such as organizing inflammation or organizing abscesses. Cases that harbored usual type hyperplasia (UTP), atypical ductal hyperplasia (ADH), or any grade of lobular neoplasia were excluded from the study.

In the malignant group, carcinomas other than (NST) ductal carcinomas, cases with incomplete hormonal immunohistochemical status, and cases for which representative tumor blocks were not available in the files of the department were all excluded from the study. Carcinomas other than (NST) ductal carcinomas were excluded from the study in order to reduce the bias of variability attributed to tumor type and hence to have as uniform an immunoexpression result as possible within the malignant group.

### 2.1. Immunohistochemistry

5 *μ*m sections were cut and mounted on saline-coated slides, dried, deparaffinized in xylene, and rehydrated in alcohol. Endogenous peroxidase was quenched by 3% hydrogen peroxidase for 10 minutes followed by heat antigen retrieval in 0.01 M citrate buffer, pH 6.0, in a microwave oven at a medium oven setting. Slides were incubated overnight at 4°C with mouse monoclonal [3D5] to glucocorticoid receptor (ab9568) (Abcam, UK) in 1 : 100 dilution. Immunoreaction was detected and visualized by using a DakoCytomation LSAB2 System (HRP code K0673). Positive cases were determined by exhibiting a focal or diffuse moderate to dark brown nuclear staining pattern in more than 10% of the cells. The percentage of cases showing positive GR expression was calculated for various categories of mammary epithelium.

Other parameters were extracted from the pathology reports of the cases such as tumor grade, ER, PR, and HER-2 expression, and axillary lymph node (ALN) status. IHC for ER and PR were determined by the Histoscore method (H score). Positivity ranged from 20/300 to 300/300. HER-2 was determined by scoring membranous staining as negative (0/1+), equivocal (2+), or positive (3+).

GR expression was compared between the malignant and the lactational groups and the malignant and the nonmalignant groups. Within the malignant group, GR expression was further analyzed according to grade, ER, PR, HER-2, or axillary lymph node (ALN) status. Analysis of GR expression according to HER-2 status excluded cases that showed HER-2 equivocal results by IHC.

Data was analyzed by using the Statistical Package for the Social Sciences (SPSS), version 15. Data was grouped into categories and analyzed for correlations using Pearson's Chi-Square test.

## 3. Results

**Figure 1:**
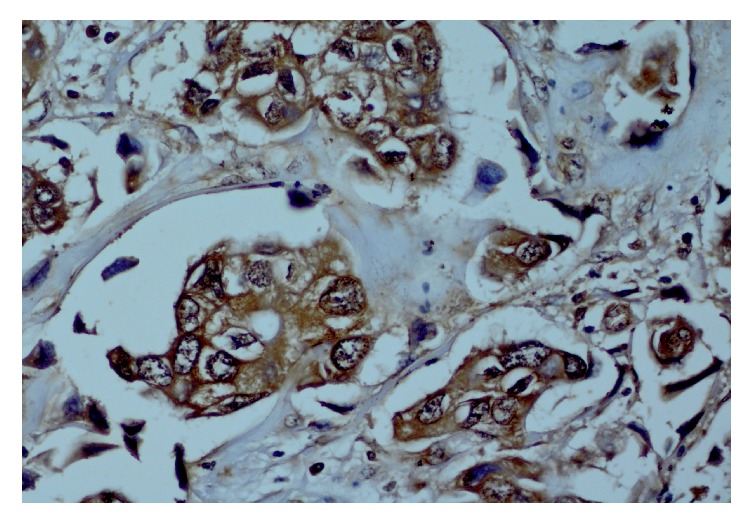
Infiltrating NST (ductal) carcinoma cells with granular brown staining of the nuclei. Background brown staining of the cytoplasm is also noted. IHC of GR (×400).

**Figure 2:**
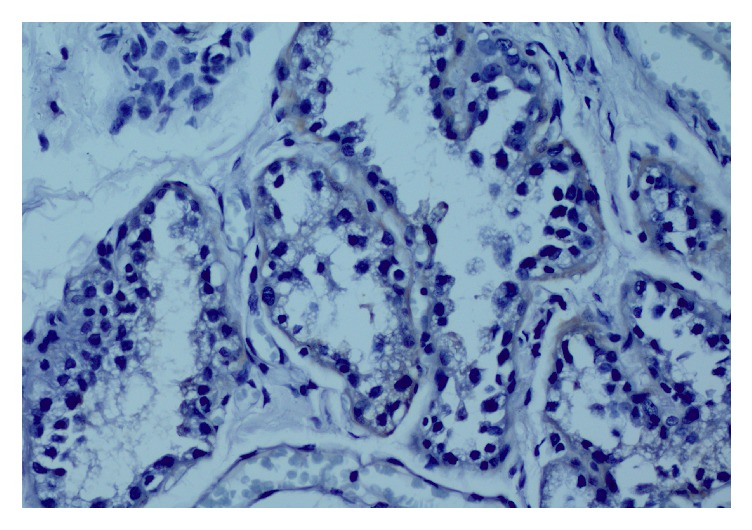
Negative staining for GR in lactational epithelium. IHC of GR (×400).

The immunoexpression of GR among the malignant group was significantly higher than that of the normal and lactational groups (*p* < 0.001) (Figures 1 and 2 and Table 1). When both the normal and the lactational groups were combined and compared together with the malignant group, statistical significance of GR expression was maintained (*p* < 0.001) (Table 2). In addition, a statistically significant correlation was noted between the GR expression and the HER-2 status as 92% of the GR-positive samples were HER-2-negative and the remaining 8% were HER-2-positive. GR expression appears to be higher in the HER-2-negative compared to the HER-2-positive tumors (*p* value: 0.002) (Table 3). Of the HER-2-negative tumors, 25 cases (69.44%) were also negative for both ER and PR (triple negative). No statistically significant difference was noted for the expression of GR in tumors categorized according to grade, ER, PR, or ALN status (*p* values: 0.331, 0.322, 0.246, and 0.517, resp.).

## 4. Discussion

Glucocorticoid receptor (GR) is a steroid hormone receptor known to influence many metabolic processes in the body including mammary development and differentiation. It is one of many factors that influences proliferation and differentiation of mammary epithelium during pregnancy and lactation. Its exact role however in carcinogenesis needs further delineation [3].

The results of this study reveal an overexpression of GR receptors in mammary malignant epithelium compared to nonmalignant tissue. This is in line with other studies [1, 2]. The action of GCs on the prepartum proliferative stage, rather than the late postpartum lactogenic stage [8], could explain the lack of overexpression of GRs in lactating epithelium in this study.

Our study is unique in that it is the first study to compare GR expression in lactating epithelium versus malignant and benign epithelium. The proliferative influence of GCs on mammary epithelial cells might explain GR overexpression in malignant mammary epithelium (the least differentiated tissue) as compared to the lack of overexpression in both lactating epithelium (most differentiated) and normal epithelium (“resting” stage).

Previous studies on mammary epithelium have shown that lobules in the lactation period of the mammary gland represent the maximal expression of development and differentiation [11, 12], hence with the least susceptibility to the influence of proliferative hormones such as GC. This further explains our results.

Other studies have implicated an antiapoptotic effect as a possible mechanism of the influence of GCs on breast cancer [13–15], affecting its initiation and progression [16, 17]. Others suggested that GCs can attenuate estrogen responses, but the mechanism by which GCs inhibit estrogenic activity is unknown. It was suggested that activation of GR by dexamethasone induces the expression and activity of estrogen sulfotransferase, an enzyme important for the metabolic deactivation of estrogen. This may have implications in therapeutic development for breast cancer [18].

Studies in the literature point to the implication of GCs in cancer progression, particularly breast cancer. An operational glucocorticoid receptor system in breast tissue was found to influence breast cancer development [19–21]. This lends additional support to the results of our study.

Other studies detailing the relationship between GCs and breast cancer included those that explored the importance of stress and its association with cancer progression, particularly breast cancer. These studies indicated that stress enhances glucocorticoid synthesis, which alters inflammation and immune responses, as well as cellular proliferation and apoptosis in a number of tissues [19–21]. In addition, activating GR-mediated tumor cell survival pathways may occur following the administration of synthetic glucocorticoids as part of chemotherapy treatment premedication, and this has the potential to diminish chemotherapy's effectiveness. Hence, the study of potential selective GR modulators may be of benefit in preventing chemotherapy associated side effects without promoting cell survival [1, 4, 13, 22–25]. In our study, we did not attempt to explore the association of GR-positive breast cancer and the response of those patients to steroid-based prechemotherapy medications, as such data was not readily available. This could be the subject of future studies to determine the level of caution that is needed, if any, in the use of steroid therapy in such category of patients.

Our study also showed GR overexpression in HER-2-negative as compared to HER-2-positive breast cancers, of which 69.44% were triple negative. This is supported by other studies which showed GR overexpression in triple negative breast cancer (TNBC) [26, 27]. This might explain chemoresistance in this group, thus opening a potential window for a different therapeutic strategy such as GR antagonists in a specific subgroup of patients such as chemotherapy-resistant GR-positive TNBC [27, 28]. GC was also found to possess a potent survival pathway in the immortalized human mammary epithelial cell line MCF10A. The mechanism through which GC inhibits apoptosis is independent of phosphatidylinositol 3-kinase activity and its downstream target Akt, thus establishing the existence of a novel epithelial cell survival pathway mediated by GCs [14].

GR immunoexpression in tumors categorized according to grade, ER, PR, or ALN status showed no statistical difference in our study. A similar study by Buxant et al. however showed a significant correlation between the histologic grade and the GR immunoexpression where the latter decreased significantly with increasing tumor histologic grade [29].

In ER-positive breast cancer, it seems that GR positivity imparts a tumor suppressor effect [30] with resultant better prognosis [31, 32]. Further immunohistochemical studies are needed to explore these relationships as studies available currently deploy other methodologies such as those studies carried out by Smith et al. where they found that GR significantly increases mRNA levels in the stroma of estrogen receptor negative tumors and an inverse relationship between sex steroid hormone receptor and GR gene expression in human breast cancer cell lines, respectively [33, 34]. Furthermore, Kinyamu and Archer showed that cross talk between the GR and ER involves multiple signaling pathways indicative of the mechanistic diversity within steroid receptor-regulated transcription [35].

Studies also showed that estrogen increases the expression of protein phosphatase 5 (PP5), which mediates the dephosphorylation of GR at Ser-211. After PP5 knockdown, estrogen-promoted cell proliferation was significantly suppressed by glucocorticoids. Thus, PP5 inhibition may antagonize estrogen-promoted events in response to corticosteroid therapy. This is supported by the fact that the course of some inflammatory diseases tends to be more severe and less responsive to corticosteroid treatment in females [36].

In our study, no attempt was made to correlate lactational changes with history of pregnancy, lactation, or duration of lactation as this study concentrated merely on cellular level changes in lactational epithelia versus other types of epithelia as explained above. This is one of the limitations of this study; however, since the results of this study show lack of overexpression of GRs in lactational change epithelium, further studies concentrating on mammary carcinoma rather than lactational change are recommended. Another limitation of this study is that the malignant group comprised NST (ductal) carcinoma cases only. Subsequent studies could concentrate on delineating GR immunoexpression in a range of breast cancer types.

Further studies are also required to support the value of including GR expression in the algorithm of breast cancer testing, to test whether ER+ GR+ tumors have better prognosis and to test whether ER− GR+ tumors are more likely to develop chemoresistance and hence might benefit from anti-GR therapy.

## 5. Conclusion

Lactational epithelium did not show overexpression of GR, while GR was overexpressed in mammary NST (ductal) carcinoma, particularly HER-2-negative cancers. Further studies are required to explore the possibility of using such receptors as targets for the development of therapeutic interventions.

## Figures and Tables

**Table 1:** Immunoexpression of GR in various categories of mammary epithelium.

Category	GR status		Total	% positivity	*p* value
	Positive	Negative			
Malignant	51	1	52	98.0%	<0.001
Normal	10	13	23	43.5%	
Lactational	12	9	21	57.1%	

**Table 2:** Immunoexpression of GR in malignant versus nonmalignant mammary epithelium.

Category	GR status		Total	% positivity	*p* value
	Positive	Negative			
Malignant	51	1	52	98.0%	<0.001
Nonmalignant	22	22	44	50.0%	

**Table 3:** Immunoexpression of GR in the malignant mammary epithelium according to HER-2 status.

Category	GR status		Total	*p* value
	Positive	Negative		
Positive HER-2	3	1	4	0.002
Negative HER-2	36	0	36	
Total	39	1	40	
